# Dipeptide repeat (DPR) pathology in the skeletal muscle of ALS patients with *C9ORF72* repeat expansion

**DOI:** 10.1007/s00401-019-02050-8

**Published:** 2019-08-02

**Authors:** Matthew D. Cykowski, Dennis W. Dickson, Suzanne Z. Powell, Anithachristy S. Arumanayagam, Andreana L. Rivera, Stanley H. Appel

**Affiliations:** 1 Department of Pathology and Genomic Medicine, Houston Methodist Hospital, 6565 Fannin St., Houston, TX 77030 USA; 2 Institute of Academic Medicine at the Houston Methodist Research Institute, Houston Methodist Hospital, 6565 Fannin St, Houston, TX 77030 USA; 3 Department of Neuroscience, Mayo Clinic, 4500 San Pablo Rd., Jacksonville, FL 32224 USA; 4 Houston Methodist Neurological Institute, Houston Methodist Hospital, 6565 Fannin St., Houston, TX 77030 USA; 5 Stanley H. Appel Department of Neurology, Houston Methodist Hospital, 6565 Fannin St., Houston, TX 77030 USA

*C9ORF72* expansion is the most common genetic alteration in both familial and sporadic ALS [9]. Dipeptide repeat (DPR) proteins are generated from repeat-associated non-ATG (RAN) translation of mutant *C9ORF72* transcripts and may be toxic to cells [4, 14] in addition to toxicity resulting from RNA foci [13]. DPRs in postmortem tissue are a pathologic hallmark of *C9ORF72*-associated amyotrophic lateral sclerosis (C9ALS), being identified in small neurons of hippocampus and cerebellum, as well as in neocortical neurons [8] and rarely in motor neurons [6]. DPRs may be identified by antibodies to poly-glycine–alanine (GA) (generated via sense translation), as well as to poly-glycine–proline (GP) (sense and antisense) and glycine–arginine (GR) (sense). Poly-GA and poly-GP inclusions are the most abundant DPRs in postmortem tissue [8] and are also recognized by p62 and ubiquitin [10].

Several studies have also suggested that DPRs are not limited to neurons. One study of human tissue, applying antibodies against GGGGCC RAN-translated peptides, showed DPRs predominantly in neurons, but also in Sertoli cells [1]. Subsequent work showed DPRs in C9ALS patient ependymal cells [10]. A recent study of a zebrafish *C9ORF72* model demonstrated DPRs in skeletal muscle [11], as well as motor neuron loss and muscle atrophy. Similarly, a transgenic fly *C9ORF72* model showed distinct perinuclear poly-GP inclusions in muscle [3]. This suggests that DPR pathology in skeletal muscle may also contribute to the ALS phenotype in disease models. It is not known whether DPR pathology is present in human ALS skeletal muscle, and thus potentially a contributing factor in C9ALS pathogenesis.

To address this question, we examined DPR inclusion pathology in 68 C9ALS skeletal muscle samples obtained from autopsies at Houston Methodist Hospital (HMH) and Mayo Clinic Jacksonville (MCJ). We assessed samples for the most common DPRs in human disease (poly-GA, poly-GP), poly-GR, as well as p62, N-terminal TDP-43, and phospho-TDP-43 (pTDP-43) pathology, using previously described methods [2, 5] (please also see Supplemental file for complete Methods). We also examined whether inclusion pathologies were associated with salient disease features, including the degree of muscle fiber atrophy, age at patient death, and disease duration.

DPRs were identified in 28 of 68 samples (41.2%) from 18 of 37 patients (48.6%). Figure 1 shows representative poly-GA, poly-GP, and p62 images from patient samples from MCJ (ALS02, ALS05) and HMH (ALS28, ALS35). DPR inclusions were comparable in size and shape to those in small neurons of dentate gyrus. DPRs were typically perinuclear and most conspicuous in atrophic fibers and were revealed by both poly-GA and poly-GP immunohistochemistry. Poly-GR immunohistochemistry, performed in a subset of 18 cases, was negative. Figure 1d, g, and j show MCJ staining with the remaining studies shown performed at HMH. Rare DPRs appeared intranuclear (please see Supplemental Fig. 2). To assess inclusion density, ten representative poly-GA-positive samples were chosen with conspicuous inclusion pathology on initial review. In these samples, inclusions were present in a median of two myofibers per 200 × field of maximal DPR pathology (IQR, 3.5 and a range of 1–15 affected fibers). Protein blotting showed poly-GA in C9ALS muscle but not in muscle samples of ALS patients without C9ORF72 expansion or non-ALS samples from inclusion body myositis (IBM). Poly-GR protein blotting, as with immunohistochemistry, was negative in C9ALS and control samples. Poly-GA immunohistochemistry was negative in five non-C9ALS muscles (two ALS patients without *C9ORF72* expansion, two IBM samples, and one neurogenic atrophy sample). Ultrastructural evaluation of a DPR-positive sample showed homogeneous intranuclear material and haphazardly arranged tubule-like structures in close proximity to subsarcolemmal nuclei. These ultrastructural findings were not seen in non-C9ALS postmortem muscle samples; however, DPRs were too sparse to definitively identify without immunoelectron microscopy, which will be a focus of future studies. Phospho-TDP-43 inclusions, also shown by N-terminal TDP-43, were present in 22 samples (32.4%) from 17 patients (46.0%) and co-localized with p62 (see Supplemental Fig. 1). TDP-43-positive foci were uniformly negative for phospho-tau and beta-amyloid and their ultrastructural correlates have previously been shown [2]. Non-muscle tissue components (e.g., nerve) was negative for pTDP-43, p62, and DPR pathology in all samples. Pathologic, demographic, and summary data are available in Supplemental Tables 1–3.

**Fig. 1:**
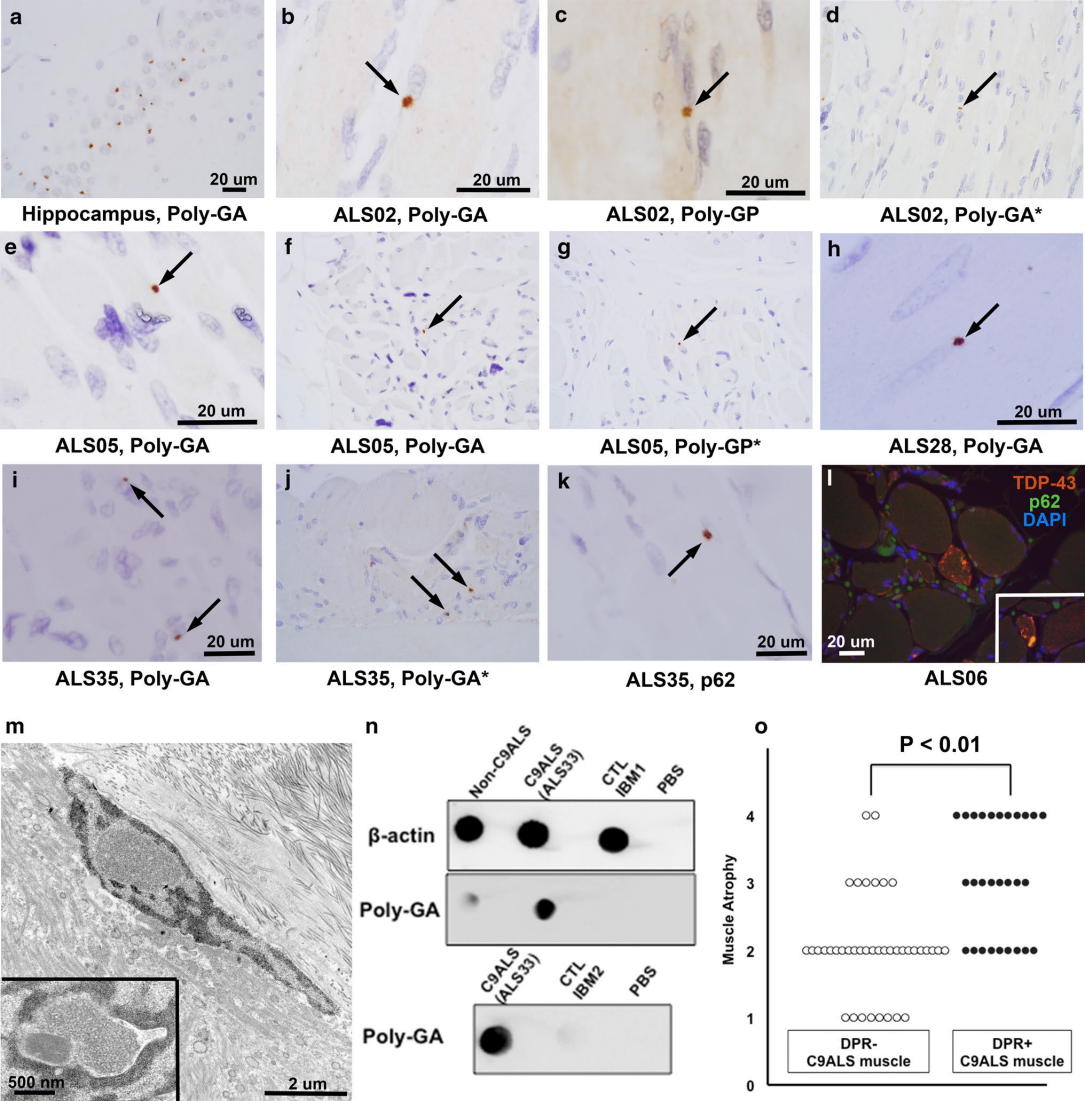
DPR pathology in postmortem C9ALS muscle samples. **a** Poly-GA staining in the dentate gyrus of a C9ALS patient. **b-j** Poly-GA and poly-GP immunohistochemistry reveals discrete, perinuclear DPR inclusions (black arrows) in muscle samples of two patients from MCJ (**b**–**g**) and two from HMH (**h**–**j**). Staining shown performed at both HMH and MCJ (latter examples are indicated by asterisks). **k** p62 perinuclear inclusion in case ALS35, which also had conspicuous poly-GA and poly-GP inclusions. **l** TDP-43 and p62-positive foci in a C9ALS muscle (inset shows an additional focus in the same patient), shown here by double-labeling immunofluorescence. Supplemental Fig. 1 shows separate color channels in two samples. **m** Electron microscopy of a DPR-positive sample showed nuclei with homogeneous, granular intranuclear material as well as tubule-like structures in close proximity to subsarcolemmal nuclei (inset). **n** Dot blot (top) of ALS without *C9ORF72* expansion (labeled “Non-C9ALS”) (left), C9ALS (middle), and control/ IBM muscle (right) showed poly-GA in C9ALS but not other samples. Repeat dot blot (bottom) in C9ALS versus an additional control sample showed the same. **o** DPR-positive C9ALS muscle samples were associated with significantly greater muscle atrophy than DPR-negative samples. For **a**–**l** all images were photographed with a 60 × objective. The scale bar in **a** applies to **d**, **f**, **g**, and **j**, which are not enlarged. **b**, **c**, **e**, **h**, **i**, **k**, and **l** are enlarged for clarity and each has an image appropriate scale bar

DPRs were significantly associated with more severe atrophy in samples (Wilcoxon rank-sum *Z* = 4.4, *P* = 1.12E − 05) (see Supplemental Table 2). DPR pathology did not significantly associate with age at death, disease duration, or pTDP-43 pathology. Among samples with any inclusion pathology, 40% of patients had DPRs and pTDP-43, 32% had DPRs only, and 28% had pTDP-43 only. DPR-positive muscle samples were from patients with lower extremity (44.4%), upper extremity (22.2%) and bulbar (22.2%) onset sites. pTDP-43-positive and negative muscle samples did not significantly differ with respect to disease duration (*P* = 0.37), though a trend was seen for pTDP-43 pathology in younger C9ALS patients (*P* = 0.06). DPR-positive and pTDP-43-positive samples did not significantly differ in frequency by axial muscle group.

These results expand the spectrum of dipeptide repeat pathology in C9ALS to include patient skeletal muscle. Significantly greater atrophy was identified in muscle samples with poly-GA and poly-GP inclusions, suggesting a potential contribution of DPRs to C9ALS muscle pathology. Poly-GR inclusions were not seen. These may be absent in C9ALS muscle, or as in brain, are too sparse in muscle to be identified without further sampling (poly-GA and poly-GP inclusions themselves were sparse). RAN-translated peptides were not seen in three patient muscle samples in a prior study [1]. A separate case report of a C9ALS patient muscle biopsy reported pleomorphic, ubiquitin and p62-positive cytoplasmic inclusions in muscle fibers [15] that more closely resemble the cytoplasmic p62 and pTDP-43-positive inclusions demonstrated here and shown previously [2]. Although this is the first demonstration of DPRs in C9ALS patient muscle, earlier studies have shown DPRs in the skeletal muscle of transgenic zebrafish [11] and fly models of C9ALS [3]. RNA foci have also been shown in iPSC-derived skeletal muscle from patients with *C9ORF72* expansion [12]. Future studies are needed to determine whether skeletal muscle-restricted expression of mutant *C9ORF72* transcripts is sufficient to drive an ALS phenotype, and if so, whether there is a beneficial response in these models to antisense oligonucleotide therapy [7].

## Electronic supplementary material

Below is the link to the electronic supplementary material.

Supplementary file1 (PDF 960 kb)
