# Supplementary material for: Dipeptide repeat (DPR) pathology in the skeletal muscle of ALS patients with C9ORF72 repeat expansion
Source: Acta Neuropathol. 2019 Aug 2;138(4):667–70. doi: 10.1007/s00401-019-02050-8 (PMC6778061; doi:10.1007/s00401-019-02050-8)
Supplement: Supplementary file 1 — Supplementary file1 (PDF 960 kb) [file 401_2019_2050_MOESM1_ESM.pdf]

Online Resource 1

Dipeptide repeat (DPR) pathology in the skeletal muscle of ALS patients with *C9ORF72* repeat expansion

Matthew D. Cykowski, Dennis W. Dickson, Suzanne Z. Powell, Anithachristy S. Arumanayagam, Andreana L. Rivera, and Stanley H. Appel

Contents:

a. Supplemental Table 1. *C9ORF72* muscle samples: Patient demographics, DPRs, and pTDP-43 pathology.

b. Supplemental Table 2. *C9ORF72* muscle samples: Pathologic findings on H&E-stained slides.

c. Supplemental Table 3. Summary of demographic and pathologic data for *C9ORF72* muscle samples.

d. Supplemental Figure 1. pTDP-43 and p62 in C9ALS muscle samples.

e. Supplemental Figure 2. Intranuclear and perinuclear inclusions with poly-GA IHC.

f. Methods.

Supplemental Table 1. *C9ORF72* muscle samples: Patient demographics, DPRs, and pTDP-43 pathology.

Study ID	Site	Age, Sex	Duration	Onset	Muscle	Poly-GA	Poly-GP	pTDP	AT8	β-amy
ALS01	MCJ	58, M	7	BUE	IP/ DPG	+/++*	0/+	+/+	0/0	0/0
ALS02	MCJ	61, M	4	RUE	IP/ DPG	++*/++	++/++	0/+	0/0	0/0
ALS03	MCJ	68, M	8	BUE	IP/DPG	0/0	0/0	0/0	0/0	0/0
ALS04	MCJ	41, F	1	RLE	IP/IP	++*/0	+/0	+/0	0/0	0/0
ALS05	MCJ	49, F	4	BLE	IP	+ +*	+	+	0/0	0/0
ALS06	MCJ	68, F	3	LUE	IP	+	0	+	0/0	0/0
ALS07	MCJ	67, M	3	RUE	IP/DPG	0/0	0/0	0/0	0/0	0/0
ALS08	MCJ	53, M	5	Bulb	IP/DPG	0/0	0/0	++/++	0/0	0/0
ALS09	MCJ	68, F	2	Bulb	IP/DPG	0/0	0/0	0/0	0/0	0/0
ALS10	MCJ	67, F	4	Bulb	IP/DPG	+/++*	+/+	0/0	0/0	0/0
ALS11	MCJ	72, M	2	LLE	IP/DPG	0/0	0/0	0/0	0/0	0/0
ALS12	MCJ	66, F	2	Bulb	IP/DPG	0/0	0/0	0/0	0/0	0/0
ALS13	MCJ	60, M	2	RUE	IP/DPG	0/0	0/0	0/0	0/0	0/0
ALS14	MCJ	71, F	3	LLE	IP/DPG	0/+	0/0	0/0	0/0	0/0
ALS15	MCJ	58, F	3	LUE	IP/DPG	0/0	0/0	0/0	0/0	0/0
ALS16	MCJ	77, F	1	Bulb	IP/DPG	0/0	0/0	0/+	0/0	0/0
ALS17	MCJ	63, M	2	RUE/gait	IP/DPG	0/0	0/0	0/0	0/0	0/0
ALS18	MCJ	61, F	1	RLE	IP	0	0	0	0	0
ALS19	MCJ	59, M	3	RLE	IP/DPG	0	0	0	0	0
ALS20	MCJ	62, F	2	RLE	IP/DPG	+/0	0/0	0/0	0/0	0/0
ALS21	MCJ	58, M	2	RUE	IP	0	0	+	0	0
ALS22	HMH	65, M	2	Limb, NOS	PS	+ +*	+	0	0	0
ALS23	HMH	61, F	1	RUE/LE, Bulb	PS/PS	0/0	0/0	0/0	0/0	0/0
ALS24	HMH	57, M	2	Bulb	PS	0	0	+	0	0
ALS25	HMH	39, M	2	LE	PS/PS	++*/++	++/+	0/++	0/0	0/0
ALS26	HMH	61, M	1	UE/LE	PS/DPG	+/+	0/0	0/0	0/0	0/0
ALS27	HMH	55, M	3	UE/LE	PS/DPG	0/0	0/0	+/+	0/0	0/0
ALS28	HMH	62, M	3	LE	PS/DPG	0/++*	0/++	+/0	0/0	0/0
ALS29	HMH	59, F	3	UE	PS/DPG	0/0	0/0	+/+	0/0	0/0
ALS30	HMH	50, F	3	LE	PS/DPG	+/+	+/0	0/0	0/0	0/0
ALS31	HMH	72, F	2	UE	PS/DPG	0/0	0/0	0/0	0/0	0/0
ALS32	HMH	68, M	2	LE	PS/DPG	+/+	0/0	0/0	0/0	0/0
ALS33	HMH	63, F	2	Bulb	PS/DPG	+ +/+ +*	+/+	++/0	0/0	0/0
ALS34	HMH	64, M	2	UE	PS/DPG	0/0	0/0	+/0	0/0	0/0
ALS35	HMH	76, F	2	Bulb	PS/DPG	++*/++	+/+	+/+	0/0	0/0
ALS36	HMH	66, F	5	Bulb	PS/DPG	0/+	0/+	0/0	0/0	0/0
ALS37	HMH	67, F	11	RUE	PS/DPG	+/+	0/0	+/0	0/0	0/0

Abbreviations and Notes: B = Bilateral; Bulb = Bulbar; DPG = Diaphragm; F = Female; HMH = Houston Methodist Hospital; IP = Iliopsoas; LE = Lower extremity; M = Male; MCJ = Mayo Clinic Jacksonville; NOS = Not otherwise specified; PS = Paraspinous; UE = Upper extremity. For IHC results, 0 = negative, + = rare inclusions on slide, ++ = conspicuous inclusions on slide. Muscle groups are separated by a forward slash for all IHC. E.g., IP/DPG with poly-GA +/++ means "rare poly-GA inclusions in iliopsoas, conspicuous inclusions in diaphragm". Asterisks in Poly-GA column indicate those samples used for semi-quantitation of inclusion density as reported in the manuscript.

Supplemental Table 2. *C9ORF72* muscle samples: Pathologic findings on H&E-stained slides.

Study ID	Muscle	Atrophy	Fibrosis	Lymph	TFs	Deg	Reg	Necrosis	Intern nuc	Other
ALS01	IP/DPG	2/2	0/0	0/0	1/0	1/1	0/0	0/0	0/0	
ALS02	IP/DPG	4/4	0/0	0/0	1/1	0/0	1/0	0/0	1/0	
ALS03	IP/DPG	2/2	0/0	0/0	0/0	0/0	0/0	0/0	0/0	
ALS04	IP/IP	2/2	1/1	1/1	0/0	1/1	1/1	1/1	0/0	Myopathic
ALS05	IP	3	1	1	1	1	1	0	1	
ALS06	IP	2	0	0	1	0	1	0	1	
ALS07	IP/DPG	4/2	0/1	0/0	0/0	0/0	0/0	0/0	0/1	Intern Nuc++
ALS08	IP/DPG	2/2	0/0	0/0	0/0	0/0	0/0	0/0	0/0	
ALS09	IP/DPG	3/2	0/0	0/0	1/1	0/0	0/0	0/0	0/0	
ALS10	IP/DPG	3/2	0/0	0/0	1/1	0/0	0/0	0/0	0/0	
ALS11	IP/DPG	2/1	0/0	0/0	1/1	0/0	0/0	0/0	0/0	
ALS12	IP/DPG	1/ 2	0/0	0/0	0/1	0/1	0/1	0/0	0/0	HFs
ALS13	IP/DPG	2/1	0/0	0/0	0/1	0/1	0/0	0/0	0/0	HFs
ALS14	IP/DPG	2/2	0/0	1/1	1/1	0/0	1/1	0/0	0/0	
ALS15	IP/DPG	2/1	0/0	0/0	0/0	0/0	0/0	0/0	0/0	
ALS16	IP/DPG	4/2	0/0	1/0	0/1	0/0	0/0	0/0	0/0	
ALS17	IP/DPG	2/1	0/0	0/0	0/1	0/0	0/0	0/0	0/0	
ALS18	IP	1	0	0	1	0	0	0	0	
ALS19	IP/DPG	3/2	0/0	0/0	1/1	0/0	0/0	0/0	0/0	
ALS20	IP/DPG	3/1	0/0	0/0	1/0	0/0	0/0	0/0	0/0	Acute inflamm
ALS21	IP	2	0	0	1	0	0	0	0	
ALS22	PS	3	0	0	1	0	0	0	0	
ALS23	PS/PS	3/2	0/0	0/0	0/0	0/0	0/0	0/0	0/0	
ALS24	PS	1	0	0	0	0	0	0	0	
ALS25	PS/PS	4/4	0/0	1/1	0/0	0/0	0/0	0/0	0/0	
ALS26	PS/DPG	4/4	0/0	0/0	0/0	0/0	0/0	0/0	0/0	
ALS27	PS/DPG	2/2	0/1	0/1	0/1	0/0	0/0	0/0	0/0	
ALS28	PS/DPG	3/3	0/0	0/0	1/1	0/1	0/0	0/0	0/0	
ALS29	PS/DPG	2/2	0/0	0/0	1/1	1/0	0/1	0/0	1/0	Intern Nuc++
ALS30	PS/DPG	4/4	1/1	0/0	0/0	0/0	0/0	0/0	0/0	
ALS31	PS/DPG	3/2	1/1	0/0	0/0	0/0	0/0	0/0	0/0	
ALS32	PS/DPG	4/3	1/0	1/0	0/0	0/1	0/0	0/0	0/0	
ALS33	PS/DPG	4/3	1/1	0/0	1/1	0/0	0/0	0/0	0/0	
ALS34	PS/DPG	3/2	0/0	1/1	0/1	1/1	1/0	0/0	0/0	Phagocytosis
ALS35	PS/DPG	2/2	0/0	0/0	1/1	1/0	1/1	0/0	0/0	
ALS36	PS/DPG	2/2	0/0	0/1	0/1	0/0	0/0	0/0	0/0	
ALS37	PS/DPG	3/4	0/0	0/0	0/0	0/0	0/0	0/0	0/0	

Abbreviations and Notes: Deg = degenerating fibers; DPG = Diaphragm; HFs = hypertrophic fibers; Lymph = Lymphocytic inflammation; Intern nuc = internalized nuclei (>10 per 400x field); IP = Iliopsoas (Mayo samples); PS = Paraspinous; Reg = regenerating fibers; TFs = target fibers. Atrophy was judged as none (0), mild without grouped atrophy (1), moderate with either <20% (2) or 20-50% of fascicles (3) having grouped atrophy, or end-stage (4).

Supplemental Table 3. Summary of demographic and pathologic data for *C9ORF72* muscle samples.

Variable	N (%) or Median (IQR)
**Age**	62 years (IQR, 9.2)
**Disease duration (years)**	2 years (IQR, 1)
**Symptom onset**	
Lower extremity	11 patients (29.7%)
Upper extremity	12 (32.4%)
Bulbar	9 (24.3%)
Multifocal	4 (10.8%)
Limb, NOS	1 (2.7%)
**Male / Female patients**	18 (48.6%)/19 (51.6%)
**Muscle pathology**	
Positive for **DPRs**	18 patients (**48.6%**)
Iliopsoas, Parasp samples positive	16 (40%)
Diaphragm samples positive	12 (42.9%)
Positive for **pTDP-43**[1]	17 patients (**46.0%**)
Iliopsoas, Parasp samples positive	14 (35.0%)
Diaphragm samples positive	8 (28.6%)
Positive for AT8 or β-amy[2]	0 (0%)
Neurogenic atrophy	37 (100%)
Non-neurogenic muscle pathologies	
Lymphocytic infiltrate[3]	9 (24.3%)
Regenerating fibers	9 (24.3%)
Internalized nuclei (>10 per 400x)	5 (13.5%)
Frankly myopathic appearance[4]	1 (2.7%)

Notes: 1, These cases had immunoreactive inclusions with pTDP-43, N-terminal TDP-43, and p62. 2, All samples were negative for tau (AT8) and beta-amyloid inclusions. No case had features of a vacuolar myopathy, such as IBM. 3, Perivascular or endomysial lymphocytic infiltrates. 4, One sample (iliopsoas) had degenerating/regenerating fibers, fiber necrosis, endomysial fibrosis and lymphocytic infiltrate and appeared frankly myopathic.

Supplemental Figure 1. pTDP-43 and p62 in C9ALS muscle samples.

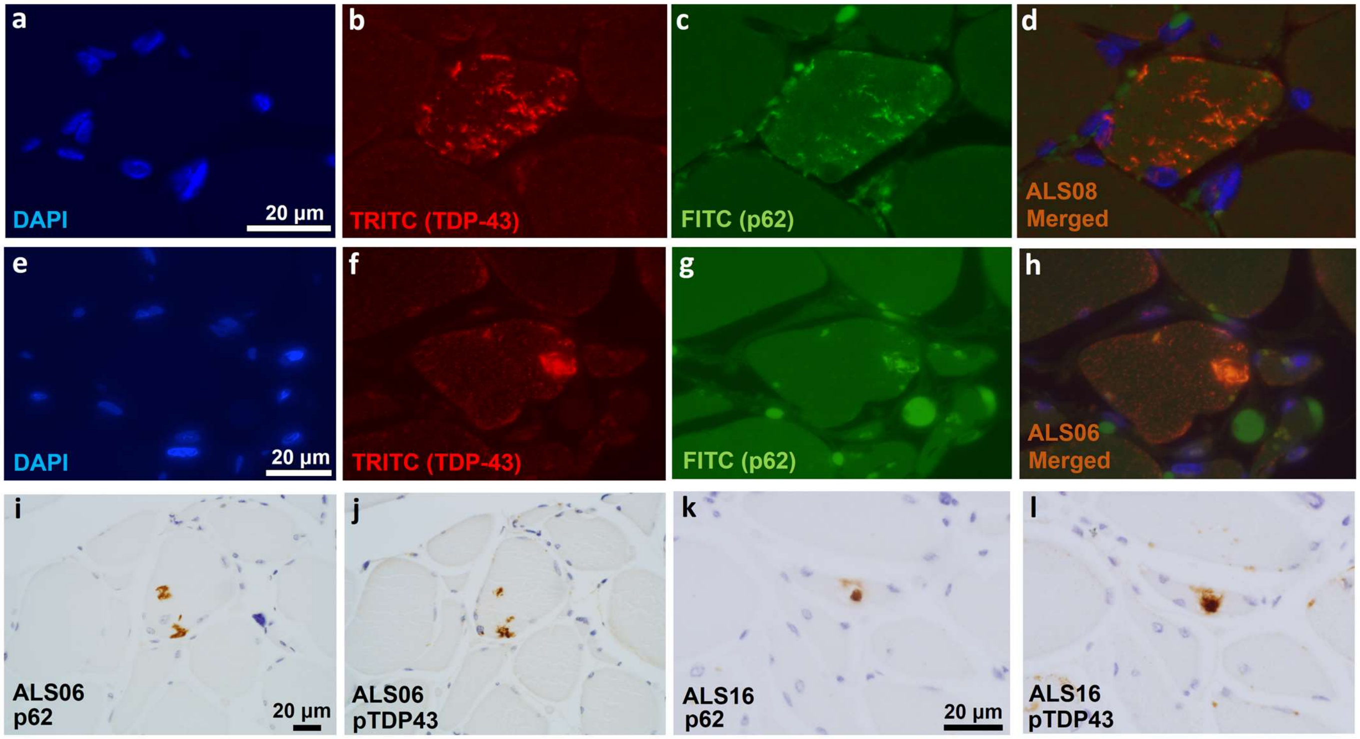

Supplemental Figure 1. DAPI (a, e), TRITC (pTDP-43) (b, f) and FITC (p62) (c, g) channel data, as well as the merged images (d, h), for two study cases from MCJ (ALS08, ALS06). Please see Supplemental Methods section for detail on immunofluorescence preparations and imaging. Merged images were generated in Olympus cellSens software from DAPI, TRITC, and FITC channel data. For panels i-l, immunohistochemical preparations on sequential five-micron sections show p62 and pTDP-43 inclusions in the same position and muscle fiber in study cases ALS06 (i, j) and ALS16 (k, l). All images taken using a 60x objective and further enlarged for detail. Scale bars in panels a, e, i, and k apply for panels a-d, e-h, i-j, and k-l, respectively.

Supplementary Figure 2. Intranuclear and perinuclear inclusions with poly-GA IHC.

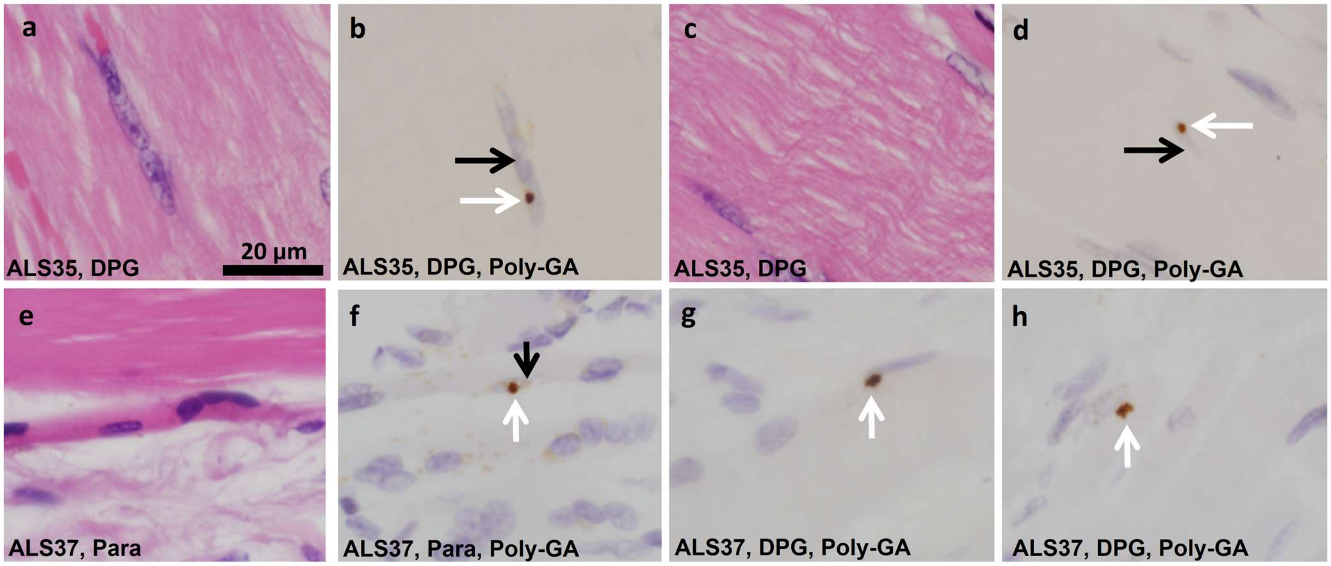

Supplemental Figure 2. Rare intranuclear (b, d, and f) inclusions with poly-GA immunohistochemistry and more typical perinuclear (g, h) inclusions as shown in two study cases from HMH (ALS35, ALS37). Samples shown are from paraspinous muscle ("Para") or diaphragm ("DPG"). All photomicrographs were taken with a 60x objective and enlarged for detail (the scale bar shown in panel a applies to all images). Black arrows in panels b, d, and f indicate the subsarcolemmal nuclei (b, f), or centrally located (d) nucleus, and white arrows in panels b, d, f, g, and h indicate the poly-GA inclusion. For approximate reference, H&E sections from the same focus in the sample are shown for panels a, c, and e, but the H&E and poly-GA IHC slides shown are not consecutive sections and are separated by two five-micron thick sections.

METHODS

Sample collection and preparation: For Houston cases, all of the ALS patients came to autopsy at Houston Methodist Hospital (HMH) and were representative of the C9ALS patients seen at our institution. No C9ALS patients were excluded. These patients were evaluated in the clinic of study author SHA and autopsies were performed by study authors MDC, SZP, or ALR. For Houston cases, autopsy blocks were, blocked, and processed after a period of fixation of one week in 20% neutral buffered formalin. Muscles routinely sampled in our autopsy protocol include paraspinous (cervical, thoracic, lumbar, and sacral levels), diaphragm, deltoid, biceps, and quadriceps. This project utilized only one paraspinous and one diaphragm sample per patient. For these anatomic sites, formalin-fixed, paraffin-embedded tissue was sectioned at 5 µm, mounted on positively charged slides, and dried at 60°C. Autopsy samples collected at Mayo Clinic Jacksonville (MCJ) were collected per their autopsy protocol under the supervision of author DWD. MCJ tissues used in this study were diaphragm and iliopsoas muscle to most closely match the axial muscle samples studied at HMH. Unstained sections of muscles of interest from MCJ were sent to the lab of study author MDC at HMH. All work on this project was performed with the approval of the Institutional Review Board at HMH (IRB 2-0114-0013).

Immunohistochemical procedures: Deparaffinization and rehydration steps were carried out using sequential washes of reagent grade xylene, graded alcohols and water. Heat-based antigen retrieval was performed using a 1X antigen retrieval solution at pH 9 (Agilent Technologies; Santa Clara, CA) for one hour (95°C for 30 min followed by 30 min on ice). All washing steps were carried out using a commercial Tris-buffered saline solution (1×) containing Tween 20, pH 7.6 (Agilent Technologies). Endogenous peroxide was blocked using a 3% hydrogen peroxide solution (VWR International; Radnor, PA). Primary antibody was applied for at least one hour at 4°C following a one hour blocking step at room temperature with 2.5% horse serum (Vector Laboratories; Burlingame, CA). Slides were thoroughly washed and the ImmPress horseradish peroxidase (HRP) anti-rabbit and anti-mouse IgG detection kits (Vector Laboratories) were applied as appropriate for 1 hour at room temperature. After additional washing steps, the target antigen was visualized using DAB chromogen in substrate buffer (Agilent Technologies). Hematoxylin counterstain was applied after additional washing steps and slides were brought to xylene and mounted with Permount (ThermoFisher Scientific; Waltham, MA).

Antibodies: Antibodies utilized against all HMH and MCJ slides included the following: p62/ Ick (mouse monoclonal, 1:100, BD Biosciences, 610833), phospho(409/410)-TDP43 (rabbit polyclonal, 1:500, Proteintech, 22309-1-AP), poly-GA (mouse monoclonal, 1:200, EMD Millipore, MABN889), poly-GP (rabbit polyclonal, 1:1000, EMD Millipore, ABN455), beta-amyloid (rabbit polyclonal, 1:2000, EMD Millipore, AB5078P, formic acid treatment performed), and phospho-tau (AT8) (mouse monoclonal, 1:1000, Thermo Fisher, MN1020). On 18 select cases (including 15 with poly-GA and/or poly-GP inclusion pathology), immunohistochemistry was also performed for poly-GR (rabbit polyclonal, 1:500, EMD Millipore, ABN1361). All immunostaining was performed with appropriate controls.

Immunofluorescence procedures: Immunofluorescence preparations were prepared in select samples for pTDP-43 and p62 co-labeling (primary review of these antibodies was done by immunohistochemistry). Slides were incubated overnight with primary antibodies at 4°C following deparaffinization, rehydration, and antigen retrieval procedures and a blocking step (2.5% horse serum), all as described above (washing steps were performed with fresh phosphate-buffered saline). Secondary antibodies were applied for 1 hour at room temperature, including Alexa Fluor 555 Anti-Rabbit IgG (1:200; A21429) and Alexa Fluor 488 anti-Mouse IgG (1:200; A11001) (Alexa Fluor are products of ThermoFisher). Primary and secondary

antibody incubation steps used combined primary and secondary antibodies in 2.5% horse serum and applied to the slide simultaneously. After additional PBS washing steps, slides were mounted using Vectashield Antifade mounting medium with 4′,6-diamidino-2-phenylindole (DAPI; Vector Laboratories). These slides were reviewed immediately and images were captured in cellSens software 1.13 (Olympus America, Inc.; Center Valley, PA) on an Olympus BX-43 Microscope using a DP71 camera, an enhanced green fluorescent protein (EGFP) FITC/Cy2 filter cube (set number 49002, Olympus; Center Valley, PA), and a CY3/tetramethylrhodamine-isothiocyanate (TRITC) filter cube (set number 49004, Olympus). Slides were examined separately under DAPI, TRITC, and FITC filters, captured, and merged in cellSens.

Protein blotting: Frozen postmortem tissue was used to extract protein using RIPA buffer (Boston Bioproducts) with between 25 and 50 mg used for extraction. 2.5 µg of protein was loaded onto a pre-wetted PVDF membrane. After incubation at RT for 30 min, the membrane was blocked using 5% non-fat dry milk (NFDM) in PBS for 30 min. Primary antibody dilutions were prepared in 5% NFDM and membranes were incubated overnight with poly-GA or poly-GR (both as above) at 4°C. After thorough washes with PBS, the membrane was incubated with appropriate anti-rabbit or anti-mouse secondary antibody conjugated to HRP and diluted in 5% NFDM for 30 min. The membrane was again thoroughly washed in PBS. Protein expression was visualized by enhanced chemiluminsecence using a Bio-Rad ChemiDoc™ MP Imaging System.

H&E muscle pathology and grading of muscle fiber atrophy: Prior to review of immunohistochemical staining, the H&E-stained sections of all muscle samples were reviewed for pathologic features. This included the presence/absence of neurogenic atrophy, myofiber necrosis, endomysial fibrosis, degeneration/regeneration, inflammation, and an increase in internalized nuclei (determined as > 10 internalized nuclei in multiple 400x fields). All muscle samples showed at least focal, mild neurogenic atrophy and were thus scored semi-quantitatively for the severity of muscle atrophy on a four-point scale. Samples were scored as mild but without grouped atrophy (score 1), moderate with fascicular atrophy in either <20% of the sample (score 2) or 20-50% of the sample (score 3), and severe/end-stage atrophy with extensive fatty infiltration (score 4). These data are shown in Supplemental Table 2.

Qualitative and semi-quantitative assessment of inclusion pathology: Upon initial pathologic review of all slides, performed using 10x, 20x, and 40x objectives by study author MDC, inclusion pathology was qualitatively recorded as absent (0), rare inclusions on the slide (+), or conspicuous inclusions on the slide (++). These values are shown for each antibody and each case in Supplemental Table 1. For poly-GA, ten representative cases were chosen with conspicuous inclusions on initial review (cases designated as ++ in Supplemental Table 1). These ten cases are indicated with an asterisk in Supplemental Table 1 under the column “Poly-GA”. For these samples, the number of positive fibers in a 200x field of maximum inclusion density was recorded.
